# Clinical characteristics of healthcare workers with SARS-CoV-2 infection after vaccination with BNT162b2 vaccine

**DOI:** 10.1186/s12879-022-07083-1

**Published:** 2022-01-28

**Authors:** Andrea Lombardi, Giulia Renisi, Dario Consonni, Massimo Oggioni, Patrizia Bono, Sara Uceda Renteria, Alessandra Piatti, Angela Cecilia Pesatori, Silvana Castaldi, Antonio Muscatello, Luciano Riboldi, Ferruccio Ceriotti, Andrea Gori, Alessandra Bandera

**Affiliations:** 1 Infectious Diseases Unit, Foundation IRCCS Ca’ Granda Ospedale Maggiore Policlinico, Milan, Italy; 2 Department of Pathophysiology and Transplantation, University of Milan, Milan, Italy; 3 Epidemiology Unit, Foundation IRCCS Ca’ Granda Ospedale Maggiore Policlinico, Milan, Italy; 4 Clinical Laboratory, Foundation IRCCS Ca’ Granda Ospedale Maggiore, Milan, Italy; 5 Medical Direction, Foundation IRCCS Ca’ Granda Ospedale Maggiore Policlinico, Milan, Italy; 6 Department of Clinical Sciences and Community Health, University of Milano, Milan, Italy; 7 Department Biomedical Sciences for Health, University of Milan, Milan, Italy; 8 Quality Unit, Foundation IRCCS Ca’ Granda Ospedale Maggiore Policlinico, Milan, Italy; 9 Occupational Health Unit, Foundation IRCCS Ca’ Granda Ospedale Maggiore Policlinico, Milan, Italy; 10 Centre for Multidisciplinary Research in Health Science (MACH), University of Milan, Milan, Italy; 11 Department of Pathophysiology and Transplantation, University of Milano Infectious Diseases Unit, IRCCS Ca’ Granda Ospedale Maggiore Policlinico Foundation, Via Francesco Sforza 35, 20122 Milan, Italy

**Keywords:** SARS-CoV-2, COVID-19, Vaccination, Symptoms, BNT162b2

## Abstract

**Background:**

The pandemic of coronavirus disease 2019 (COVID-19), caused by the severe acute respiratory syndrome coronavirus 2 (SARS-CoV-2), had a significant impact worldwide. Vaccines against COVID-19 appear as a tool able to curb out mortality and reduce the circulation of the virus. Little is known so far about the clinical characteristics of individuals who developed SARS-CoV-2 infection after having received the vaccination, as well as the temporal relationship between vaccine administration and symptoms onset.

**Methods:**

Retrospective cohort study among the 3219 healthcare workers (HCWs) of the Fondazione IRCCS Ospedale Maggiore Policlinico of Milano who received a full immunization with the BNT162b2 vaccine and who developed SARS-CoV-2 infection (documented through positive RT-PCR on nasopharyngeal swab) in March–April 2021.

**Results:**

Overall, we have identified 15 HCWs with SARS-CoV-2 infection after vaccination, 7 (46.7%) of them were male and the mean age was 38.4 years (SD 14). In 4 of them, the presence of SARS-CoV-2 anti-nucleocapsid (anti-N) antibodies was assessed before vaccination and resulted positive in 1 case. In all HCWs the presence of SARS-CoV-2 anti-spike (anti-S1) antibodies was assessed, on average 42.2 days after the completion of vaccination, with a mean value of 2055 U/mL (SD 1927.3). SARS-CoV-2 infection was ascertained on average 56.2 days after vaccination. The mean cycle threshold (Ct) of SARS-CoV-2 PCR was 26.4, the lineage was characterized in 9 HCWs. None of the HCWs reported a primary or secondary immunodeficiency. Regarding symptoms, they were reported only by 7 (46.7%) HCWs and appeared on average 55 days after the second dose of vaccination. Of those who reported symptoms, one (14.3%) had fever, 7 (100%) rhinitis/conjunctivitis, 4 (57.1%) taste and smell alterations, none had respiratory symptoms, 4 headache/arthralgia (57.1%) and 1 gastrointestinal symptom (14.3%). All symptoms disappeared in a few days and no other unclassified symptoms were reported.

**Conclusions:**

Infections occurring after vaccination with the BNT162b2 vaccine are mostly asymptomatic and are not associated with the serum titre of anti-S1 antibodies. We did not find a predominance of specific viral variants, with several lineages represented.

## Background

The pandemic of coronavirus disease 2019 (COVID-19), caused by the severe acute respiratory syndrome coronavirus 2 (SARS-CoV-2), has caused significant morbidity and mortality. Vaccines against COVID-19 appear as a tool able to curb out mortality and reduce the circulation of the virus. Consequently, mass vaccination campaigns are ongoing worldwide [1, 2]. Healthcare workers (HCWs) are a population who has been vaccinated early in the pandemic due to their high exposure to the virus, the corresponding elevated risk of infection and the possible role in spreading the disease [3].

Little is known so far about the clinical characteristics of HCWs who developed SARS-CoV-2 infection after having received the vaccination, as well as the temporal relationship between vaccine administration and symptoms onset. To answer these questions, we conducted a retrospective study among the HCWs vaccinated with BNT162b2 who developed SARS-CoV-2 infection (documented through positive RT-PCR on nasopharyngeal swab) in a large university hospital, collecting their clinical characteristics.

## Methods

All the HCWs of the IRCCS Ospedale Maggiore Policlinico, a university hospital in Milan, Italy, were offered the COVID-19 vaccination with BNT162b2 vaccine. Among the 3622 HCWs working in our centre at the time of the study, 3219 (88.9%) received the full schedule, 170 (4.7%) received only the first shot and 233 (6.4%) were not vaccinated. The two prescribed shots were administered during January and February 2021. The HCWs of our hospital who are working in direct contact with proven or possible COVID-19 patients are subject to mandatory surveillance nasopharyngeal swab (NPS) for SARS-CoV-2 every two weeks, irrespective of the presence of symptoms. We collected the demographic, clinical and virologic characteristics of those who had positive NPSs in the period 01/03/2021–30/04/2021. For SARS-CoV-2 RNA detection was used the Alinity m SARS-CoV-2 assay on Alinity m (Abbott Molecular, IL, USA). The test is an rRT-PCR that allow simultaneous detection of RdRp and N genes. Serologic analyses were performed with two electrochemiluminescence immunoassay (ECLIA), Elecsys Anti-SARS-CoV-2 and Elecsys Anti-SARS-CoV-2 Son Cobas e801 (Roche Diagnostic, Mannheim, Germany) for the detection of total antibodies (including IgG) directed against SARS-CoV-2 nucleocapsid (N) antigen and SARS-CoV-2 spike protein receptor-binding domain (RBD) respectively. Full genome sequences were obtained by amplifying using CleanPlex for SARS-CoV-2 Research and Surveillance NGS panel (Paragon Genomics, Hayward CA, USA). A library was prepared with the PCR products and sequencing was performed on the Illumina MiSeq platform. The results were aligned to the reference genome NC_045512.2 by SOPHiA DDM software, v4 (SOPHiA GENETICS, USA). The software used to assign lineages to SARS CoV-2 sequences was Phylogenetic Assignment of Named Global Outbreak LINeages (Pango COVID-19 Lineage Assigner) [4, 5]. Descriptive statistics were obtained for all the variables collected, analyses were performed with Stata 17 (StataCorp. 2019). All the enrolled patients signed written informed consent. The study protocol (#828_2021) was approved by the local (Milano Area 2) Ethics Committee.

## Results

Overall, we have identified 15 HCWs with SARS-CoV-2 infection after vaccination, 7 (46.7%) of them were male and the mean age was 38.4 years (SD 14). In 4 of them, the presence of SARS-CoV-2 anti-nucleocapsid (anti-N) antibodies was assessed before vaccination and resulted positive in 1 case. In all HCWs the presence of SARS-CoV-2 anti-spike (anti-S1) antibodies was assessed, on average 42.2 days after the completion of vaccination, with a mean value of 2055 U/mL (SD 1927.3). SARS-CoV-2 infection was ascertained on average 56.2 days after vaccination. The mean cycle threshold (Ct) of SARS-CoV-2 PCR was 26.4, the lineage was characterized in 9 HCWs (Table 1). None of the HCWs reported a primary or secondary immunodeficiency.

**Table 1:** Demographic and virologic characteristics of patients vaccinated with BNT162b2 with documented SARS-CoV-2 infection through positive RT-PCR on nasopharyngeal swab

ID	Age	Sex	Previous COVID-19	Days between vaccination and serology	Anti-S1 antibodies (U/mL)	Days between vaccination and symptoms	Days between vaccination and positive swab	Ct	Lineage
1	29	M	Unknown	24	2974	No symptoms	36	41	Unknown
2	28	F	Unknown	46	460	No symptoms	29	36.5	Unknown
3	29	M	No	82	866	26	24	21.8	B.1.351
4	28	F	Unknown	63	3787	No symptoms	29	14.2	B.1.1.241
5	60	M	Unknown	36	353	63	65	16.5	C.36
6	28	F	Unknown	35	3838	No symptoms	72	21.6	C.36
7	38	F	Unknown	37	1902	50	51	17.8	P.1
8	22	F	Unknown	65	1925	No symptoms	66	34.5	Unknown
9	46	M	No	34	637	58	58	16.6	B.1.1.7
10	31	M	Unknown	36	1493	34	35	19.8	B.1.525
11	57	M	Unknown	37	162	No symptoms	70	41.9	Unknown
12	39	F	Yes	36	> 7500	93	95	39.6	Unknown
13	66	M	No	40	1115	No symptoms	109	20.4	C.11
14	48	F	Unknown	28	2790	No symptoms	49	39.4	Unknown
15	26	F	Unknown	34	1005	55	55	14.1	B.1.177.7

*CT* cycle threshold

Regarding symptoms, they were reported only by 7 (46.7%) HCWs and appeared on average 55 days after the second dose of vaccine. Of those who reported symptoms, one (14.3%) had fever, 7 (100%) rhinitis/conjunctivitis, 4 (57.1%) taste and smell alterations, none had respiratory symptoms, 4 headache/arthralgia (57.1%) and 1 gastrointestinal symptom (14.3%). All symptoms disappeared in a few days and no other unclassified symptoms were reported (Table 2).

**Table 2:** Symptoms reported at time of positive nasopharyngeal swab in patients vaccinated with BNT162b2 with documented SARS-CoV-2 infection through positive RT-PCR

ID	Symptoms	Fever	Rhinitis/conjunctivitis	Taste/Smell alterations	Cough/dyspnoea	Headache/arthralgia	GI symptoms	Other
1	No	No	No	No	No	No	No	No
2	No	No	No	No	No	No	No	No
3	Yes	No	Yes	Yes	No	Yes	No	No
4	No	No	No	No	No	No	No	No
5	Yes	No	Yes	No	No	Yes	No	No
6	No	No	No	No	No	No	No	No
7	Yes	No	Yes	Yes	No	No	No	No
8	No	No	No	No	No	No	No	No
9	Yes	No	Yes	No	No	Yes	Yes	No
10	Yes	No	Yes	Yes	No	No	No	No
11	No	No	No	No	No	No	No	No
12	Yes	No	Yes	No	No	Yes	No	No
13	No	No	No	No	No	No	No	No
14	No	No	No	No	No	No	No	No
15	Yes	Yes	Yes	Yes	No	No	No	No

*GI* gastrointestinal

## Discussion

We have identified 15 HCWs who developed SARS-CoV-2 infection after completing the vaccination schedule with the BNT162b2 vaccine. Symptoms were reported by less than half of those included in the study, were mild, with only one case of fever, and disappeared quickly. The infections were detected through the mandatory surveillance system applied in our hospital, on average almost 2 months after the completion of the vaccination schedule.

The absence of important symptoms is a reassuring finding, which confirms the data about the efficacy of the BNT162b2 vaccine in preventing the severe form of COVID-19 reported in the registration study [6]. Intriguingly is the high incidence of rhinitis and conjunctivitis, which resulted in the most frequently reported symptom. Conjunctivitis is usually reported in about 1% of COVID-19 patients, with a higher prevalence (3%) among severe cases. The expression on the conjunctiva of the entry receptor for SARS-CoV-2, ACE-2, is disputed [7, 8], even though it has been shown how the inoculation in the ocular conjunctiva of SARS-CoV-2 can cause mild COVID-19 in rhesus macaques [9]. It is possible to speculate that the conjunctiva represents the site with the highest viral concentration in patients with the infection after vaccination, and this is reflected by the presence of a vigorous inflammatory response. Further studies are necessary to assess the concentration of immunoglobulins against SARS-CoV-2 in the ocular fluids after vaccination and their neutralizing activity.

In a previous publication from our group, we described how SARS-CoV-2 reinfections occurring in HCWs with a previous diagnosis of COVID-19 were mostly asymptomatic (7/9) [10]. Instead, we have previously observed during the first wave of COVID-19 pandemic how only a minority of HCWs has asymptomatic SARS-CoV-2 infections (17/139) [11]. This reinforces the concept of reduced/absent symptoms in those who probably have developed immunity against SARS-CoV-2, both through infection or vaccination.

Regarding the mean value of the serum anti-S1 antibodies, we observed in those who developed SARS-CoV-2 infection a value superior to the mean value of all the vaccinated HCWs of our hospital (1577 U/mL) and also superior to the mean value of the vaccinated HCWs without a previous SARS-CoV-2 infection (1374 U/mL) [12]. This suggest that the raw value of the anti-S1 antibodies cannot predict the future development of infection, the neutralizing activity of these antibodies could be a better tool to predict the efficacy of the humoral response.

Interestingly, we did not find a predominance of specific viral variants, with several lineages represented. This is in accordance with published data, which highlighted a reduced but still efficacious immune response against viral variants in those vaccinated with BNT162b2, and suggest that the risk of infection after vaccination is not currently related to the viral genotype but to other variables yet to be uncovered [1, 13]. Of note, the prevalence of SARS-CoV-2 lineages detected is adherent to the Italian scenario at the time, with the Alpha variant the most frequently reported [14].

A limitation of our study is the short observation time post-vaccination, restricted to two months, which led to the identification of only 15 infections post-vaccination in a large cohort of HCWs. A longer observation time might have led to the identification of a higher number of cases, increasing the chance of also finding infections with severe clinical manifestations.

## Conclusions

Infections occurring after vaccination with the BNT162b2 vaccine are mostly asymptomatic and are not associated with the serum titre of anti-S1 antibodies.

## Data Availability

The datasets used and/or analysed during the current study are available from the corresponding author on reasonable request.

## Abbreviations

COVID-19: Coronavirus disease 2019
SARS-CoV-2: Severe acute respiratory syndrome coronavirus 2
HCWs: Healthcare workers
ECLIA: Electrochemiluminescence immunoassay

## References

[1] Haas EJ, Angulo FJ, Mclaughlin JM, Anis E, Singer SR, Khan F (2021). Impact and effectiveness of mRNA BNT162b2 vaccine against SARS-CoV-2 infections and COVID-19 cases, hospitalisations, and deaths following a nationwide vaccination campaign in Israel: an observational study using national surveillance data. Lancet.

[2] Lombardi A, Bozzi G, Ungaro R, Villa S, Castelli V, Mangioni D (2021). Mini review immunological consequences of immunization with COVID-19 mRNA vaccines: preliminary results. Front Immunol.

[3] Lombardi A, Mangioni D, Consonni D, Cariani L, Bono P, Cantù AP (2021). Seroprevalence of anti-SARS-CoV-2 IgG among healthcare workers of a large university hospital in Milan, Lombardy, Italy: a cross-sectional study. BMJ Open.

[4] Rambaut A, Holmes EC, O’Toole Á, Hill V, McCrone JT, Ruis C (2021). Addendum: A dynamic nomenclature proposal for SARS-CoV-2 lineages to assist genomic epidemiology. Nat Microbiol.

[5] Rambaut A, Holmes EC, O’Toole Á, Hill V, McCrone JT, Ruis C (2020). A dynamic nomenclature proposal for SARS-CoV-2 lineages to assist genomic epidemiology. Nat Microbiol.

[6] Polack FP, Thomas SJ, Kitchin N, Absalon J, Gurtman A, Lockhart S (2020). Safety and efficacy of the BNT162b2 mRNA covid-19 vaccine. N Engl J Med.

[7] Zhou L, Xu Z, Castiglione GM, Soiberman US, Eberhart CG, Duh EJ (2020). ACE2 and TMPRSS2 are expressed on the human ocular surface, suggesting susceptibility to SARS-CoV-2 infection. Ocul Surf.

[8] Lange C, Wolf J, Auw-Haedrich C, Schlecht A, Boneva S, Lapp T (2020). Expression of the COVID-19 receptor ACE2 in the human conjunctiva. J Med Virol.

[9] Deng W, Bao L, Gao H, Xiang Z, Qu Y, Song Z (2020). Ocular conjunctival inoculation of SARS-CoV-2 can cause mild COVID-19 in rhesus macaques. Nat Commun.

[10] Comelli A, Consonni D, Lombardi A, Viero G, Oggioni M, Bono P (2021). Nasopharyngeal testing among healthcare workers (HCWs) of a Large University Hospital in Milan, Italy during two epidemic waves of COVID-19. Int J Environ Res Public Health.

[11] Lombardi A, Consonni D, Carugno M, Bozzi G, Mangioni D, Muscatello A (2020). Characteristics of 1573 healthcare workers who underwent nasopharyngeal swab testing for SARS-CoV-2 in Milan, Lombardy, Italy. Clin Microbiol Infect.

[12] Lombardi A, Consonni D, Oggioni M, Bono P, Renteria SU, Piatti A (2021). SARS-CoV-2 anti-spike antibody titres after vaccination with BNT162b2 in naïve and previously infected individuals. J Infect Public Health.

[13] Liu Y, Liu J, Xia H, Zhang X, Fontes-Garfias CR, Swanson KA (2021). Neutralizing activity of BNT162b2-elicited serum. N Engl J Med.

[14] Prevalenza e distribuzione delle varianti del virus SARS-CoV-2 di interesse per la sanità pubblica in Italia.

